# 1283. Safety, Tolerability, and Pharmacokinetics of BRII-778, A Modified-Release Oral Formulation of Rilpivirine in Healthy Adult Subjects

**DOI:** 10.1093/ofid/ofac492.1114

**Published:** 2022-12-15

**Authors:** David A Margolis, Ji Ma, Michael Watkins, Yujin Wang, Chetana Trivedi, Xuelian Wei, Lijie Zhong, Kamlesh Patel, Li Yan, Zhi Hong, Jean-Luc Girardet, Lianhong Xu

**Affiliations:** Brii Biosciences, Durham, NC; Brii Biosciences, Durham, NC; Brii Biosciences, Durham, NC; Brii Biosciences, Durham, NC; Brii Biosciences, Durham, NC; Brii Biosciences, Durham, NC; Brii Biosciences, Durham, NC; Brii Biosciences, Durham, NC; Brii Biosciences, Durham, NC; Brii Biosciences, Durham, NC; Brii Biosciences, Durham, NC; Brii Biosciences, Durham, NC

## Abstract

**Background:**

BRII-778 is a modified release (MR) formulation of rilpivirine for once-weekly (QW) oral administration. BRII-778 aims to prolong oral absorption, lower C_max_, relatively, and reduce peak to trough ratio achieved with a MR formulation of rilpivirine, within the known efficacy and safety bounds established by once daily oral administration of rilpivirine. QW BRII-778 dosing may have advantages over once daily rilpirvirine in terms of patient convenience, and treatment adherence. Multiple MR formulations of rilpivirine were evaluated in healthy adult subjects in this Phase 1 study.

**Methods:**

This was a Phase 1, randomized, double-blinded, placebo-controlled, study of single ascending dose (SAD) and multiple ascending dose (MAD) cohorts, evaluating the safety, tolerability, and PK of BRII-778. Three MR formulations of rilpivirine, BRII-778-A1, -A2, and -A3, were evaluated in this study. Rilpivirine PK profiles after single or multiple doses of the three BRII-778 formulations were characterized under the fed state over the dose range of 150 mg to 750 mg. Food effect was assessed after a single oral dose of BRII-778-A3 750 mg. Exploratory concentration-QTc (C-QTc) analysis was conducted using combined SAD and MAD data.

**Results:**

BRII-778 as single dose or multiple doses was generally safe and well-tolerated when administered to healthy adult subjects. There were no Grade ≥3 AEs, SAEs or AEs leading to withdrawal in BRII-778 dosing arms. PK profiles of BRII-778 were consistent with slower oral absorption with MR formulation. The increase in exposure was less than dose proportional. Mild accumulation in plasma was observed after 3 QW BRII-778-A3 doses. BRII-778-A3 750 mg under the fed state enhanced bioavailability by improving gastric dissolution and/or subsequent absorption. Exploratory C-QTc analysis confirmed a concentration-dependent effect on the QTc interval with escalating doses of BRII-778, but the interpretations are limited by small sample size. There were no clinically significant EKG changes and no individual subject met QTc stopping criteria.

**Conclusion:**

SAD and MAD administration of BRII-778 formulations were generally safe and well tolerated. Rilpivirine PK profiles post BRII-778 dosing supports further evaluation of BRII-778 for potential QW regimen.

**Disclosures:**

**David A. Margolis, MD MPH**, Brii Biosciences: Stocks/Bonds **Ji Ma, PhD**, Brii Biosciences: Stocks/Bonds **Michael Watkins, PharmD**, Brii Biosciences: Stocks/Bonds **Yujin Wang, M.Sc.**, Brii Biosciences: Stocks/Bonds **Chetana Trivedi, B.A.**, Brii Biosciences: Stocks/Bonds **Xuelian Wei, PhD**, Brii Biosciences: Stocks/Bonds **Lijie Zhong, PhD**, Brii Biosciences: Stocks/Bonds **Kamlesh Patel, PhD**, Brii Biosciences: Stocks/Bonds **Li Yan, MD PhD**, Brii Biosciences: Stocks/Bonds **Zhi Hong, PhD**, Brii Biosciences: Ownership Interest **Jean-Luc Girardet, PhD**, Brii Biosciences: Stocks/Bonds **Lianhong Xu, PhD**, Brii Biosciences: Stocks/Bonds.

